# Epidemiological analysis of hydrometra and its predictive value in gynecological tumors

**DOI:** 10.3389/fonc.2022.1028886

**Published:** 2023-01-05

**Authors:** Jianfa Wu, Sihong Wang, Li Zhang, Suqin Wu, Zhou Liu

**Affiliations:** ^1^ Department of Gynecology, Shanghai University of Medicine & Health Sciences Affiliated Zhoupu Hospital, Shanghai, China; ^2^ Department of Gynecology, Shanghai University of Medicine & Health Sciences, Shanghai, China

**Keywords:** hydrometra, endometrial cancer, cervical cancer, inflammation, risk factor

## Abstract

**Introduction:**

Hydrometra is a common gynecological disease, especially in postmenopausal women. However, its epidemiology, harmfulness, and value in predicting gynecological tumors have not been clearly elucidated.

**Methods:**

In this study, the prevalence rate of and risk factors for hydrometra were investigated in 3,903 women who underwent screening for gynecological diseases at Zhoupu Hospital in Shanghai from 1 January to 31 December 2021. In addition, pathological distribution of hydrometra and its predictive value in gynecological tumors were studied in another 186 patients in whom hydrometra was diagnosed sonographically at Zhoupu Hospital, from 1 January 2020 to 31 December 2021, and who underwent hysteroscopy and postoperative pathological examination.

**Results:**

The observed prevalence rate of hydrometra was 10.86%, which was higher than the prevalence of other gynecological diseases. Univariate and multivariate analysis indicated that advanced age (OR 1.11) and vaginitis (OR 3.18) were independent risk factors for hydrometra. Among 186 patients with a sonographic diagnosis of uterine fluid, simple hydrometra accounted for 34.41% of cases, inflammation accounted for 16.23%, and hematometra accounted for 2.15%, while gynecological tumors accounted for 5.91%. Moreover, univariate and multivariate analysis indicated that a higher body mass index (>23.92 kg/m^2^), greater hydrometra volume (i.e., distance between the two layers of endometrium>4.75 mm), and abnormal vaginal bleeding were high-risk predictive factors for gynecological tumors.

**Discussion:**

In conclusion, hydrometra is a common disease, and is a risk factor for endometrial cancer and cervical cancer, especially in patients with higher hydrometra volume, higher BMI, and abnormal vaginal bleeding. It is necessary to pay more attention to hydrometra.

## Introduction

Hydrometra is a common disease, with a reported prevalence rate of 14.1% (1). Inflammation is considered to be the most important cause, especially in postmenopausal women, and is rare in premenopausal women (2, 3). Estrogen stimulation or treatment with tamoxifen also contributes to hydrometra development (4), and obstruction of the cervix, vagina, or fallopian tubes is thought to play an important role (5). Other factors associated with high risk for hydrometra include advanced age and long postmenopausal time, while hormone replacement therapy was once thought to be a potential treatment for hydrometra (1).

Moreover, previous studies have considered hydrometra as a barrier to reproduction, accounting for reduced pregnancy rates in women undergoing *in vitro* fertilization (IVF) (5–7). However, this view remains controversial. Mehtap Polat etal. (8) reported that transient intrauterine fluid accumulation was not detrimental to IVF if it was not due to hydrosalpinx or any identifiable pelvic pathology.

Hydrometra has been considered as a typical symptom of genital tract tumors, and this is especially true of fallopian tube carcinoma, ovarian cancer, and endometrial carcinoma (9–11). Other researchers believe that hydrometra can be used as a prognostic factor in cervical cancer and endometrial carcinoma (12, 13). However, most previous studies were case reports, and systematic studies on hydrometra are still lacking. Moreover, hydrometra, being a common symptom, is usually ignored as a warning of potential adverse effects on women’s health, especially cancer. There remains a lack of large-scale epidemiological studies of hydrometra to clarify its incidence, risk factors, and value in predicting in tumors. Moreover, studies of the pathogenesis of hydrometra have been mainly carried out in animals, so risk factors for hydrometra are still unknown.

In this study, we attempted to determine the epidemiology of and risk factors for hydrometra through regional screening for gynecological diseases. Furthermore, the pathological distribution of 186 cases of hydrometra after hysteroscopy was analyzed retrospectively to determine its perniciousness. In addition, we discuss factors that determine the role of hydrometra in gynecological tumor prediction.

## Methods

### Gynecological diseases screening information

To investigate the prevalence rate and risk factors of hydrometra, we studied 4,140 women who underwent screening for gynecological diseases in Zhoupu Hospital from 1 January to 31 December 2021. Of these, 237 women were excluded from this study because they had had a hysterectomy, leaving 3,903 women included in the study. All 3,903 patients had undergone all screening tests for gynecological diseases and provided complete data for study. All gynecological disease screening was performed by senior attending physicians or more senior doctors.

Uterine fibroids, ovarian cysts, hydrometra, and endometrial lesions were certified by transvaginal ultrasound examination with a LOGIQ E9 scanner (GE, USA) using a 4- to 10-Hz transducer. The structure and thickness of the endometrium were recorded for each woman. Abnormal endometrium was defined as an endometrial thickness of > 5 mm on ultrasound in a postmenopausal woman or > 15 mm in a premenopausal woman (14). Uterine fibroids were diagnosed by ultrasound as previously described by Fascilla etal. (15). Ovarian cysts were diagnosed by ultrasound as reported by Granberg and Wikland (16) and Vitale etal. (17). Adenomyosis was diagnosed by ultrasound as described by Andres etal. (18). The volume of hydrometra was determined by measuring the distance between the two layers of endometrium.

Prolapse of the uterus was diagnosed in accordance with Guideline No. 413 (19), and was certified by two experienced gynecologists.

Cervical lesions were screened using a ThinPrep cytologic test (TCT). The results of TCTs were determined by two experienced pathologists in accordance with the 2001 Bethesda System (20). Cervical polyps were diagnosed as described previously (21).

Vaginitis was detected in accordance with the relevant American College of Obstetricians and Gynecologists (ACOG) Practice Bulletin (22). If cleanliness of leukorrhea was more than 3 degree or the presence of Trichomonas, mycetes, or clue cells was found, then vaginitis was diagnosed.

### Information on patients with hydrometra

To study the pathological outcome of hydrometra and its relationship with gynecological tumors, another resident patient sample with hydrometra was employed, this time comprising 186 patients who received a sonographic diagnosis of hydrometra in Zhoupu Hospital from 1 January 2020 to 31 December 2021. All patients were divided into two groups according to the pathological findings: a positive group (i.e., those with cervical cancer, high-grade squamous intraepithelial lesion of the cervix, atypical hyperplasia of the endometrium, or endometrial cancer) and a negative control (NC) group (i.e., those patients with none of the above lesions).

All patients underwent hysteroscopy and postoperative pathological examination by an experienced gynecologist and pathologist. Moreover, all patients underwent cervical canal and uterine cavity sampling during hysteroscopy. The presence of hydrometra was certified by hysteroscopy, the presence of an intrauterine device (IUD) was determined by hysteroscopy and ultrasound, and the presence of intrauterine occupation was determined by pathological findings. Body mass index (BMI) was calculated using the formula weight/height^2^.

### Ethics statement

The protocol for this research project was approved by Zhoupu Hospital Ethics Committee and conformed to the provisions of the Declaration of Helsinki of 1995 (as revised in Brazil, 2013). All patient information was exported from the hospital information system (HIS) electronic medical record system. All patients provided written informed consent and patient anonymity was preserved.

### Statistical methods

All statistical analyses were performed using SPSS software (version 22). Depending on the sample size, quantitative data were analyzed using the chi-squared test, the chi-squared test with continuous correction, or Fisher’s exact probability method. In the case of measurement data, the two independent-samples Student’s *t*-test, a corrected *t*-test, or the Wilcoxon rank-sum test was used to compare differences between the two groups. Multivariate analysis was performed using two-factor logistic regression analysis. Values of 0 or 1 were assigned to dichotomous variables, 0 for “no” and 1 for “yes”. For continuous variables, original data were used for the regression analyses. Differences were considered significant at a two-sided *p*-value < 0.05.

## Results

### Analysis of prevalence of hydrometra

**Figure 1:**
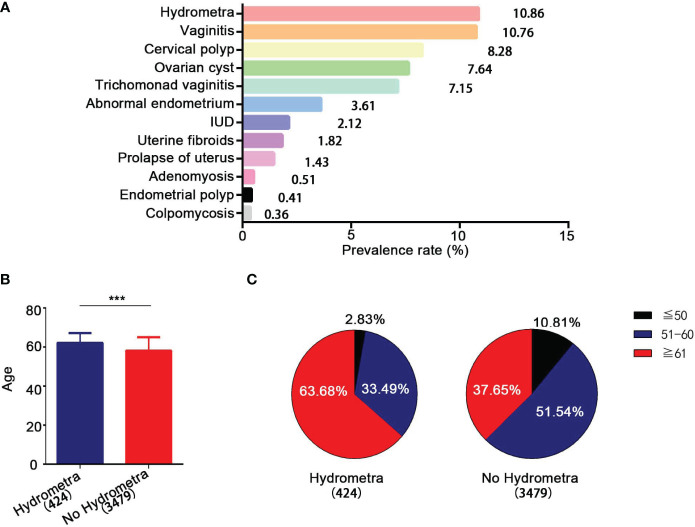
Epidemiological analysis of hydrometra. **(A)** Prevalence rate of different gynecological diseases. The *y*-axis represents different gynecological diseases, and the *x*-axis represents prevalence rate. **(B)** Age comparison between women with and without hydrometra. **(C)** Age distribution comparison between women with hydrometra and without hydrometra. ***p<0.001.

In this study, the prevalence rate of different gynecological diseases among women undergoing screening in Pudong New Area in Shanghai was determined. The clinical and demographic characteristics of the 3,903 women who participated are shown in **Table S1**. Among the 3,903 women who underwent gynecological screening, the lesion with the highest prevalence rate was hydrometra, reaching 10.86% (**Figure 1A**). This was followed by vaginitis (prevalence rate 10.76%), cervical polyps (prevalence rate 8.28%), abnormal endometrium (prevalence rate 3.61%), uterine fibroids (prevalence rate 1.82%), prolapse of uterus (prevalence rate 1.43%), and adenomyosis (prevalence rate 0.51%) (**Figure 1A**). Moreover, it was found that the mean (SD) age of women with hydrometra was higher than that of women without hydrometra (62.07 ± 0.25 years, compared with 58.12 ± 0.12 years, respectively; *p* < 0.0001; **Figure 1B**). The age distribution of women with and without hydrometra was also analyzed. It was found that the prevalence rate of hydrometra was highest in the ≥ 60 years age group (63.68%) and lowest in the ≤ 50 years age group (2.83%) (**Figure 1C**). In contrast, it was found that the prevalence rate of no hydrometra was lower in the ≥ 60 years age group (37.65%) (**Figure 1C**). These data suggest that hydrometra is a common disease in women, especially in those aged ≥ 60 years.

### Risk factors analysis for hydrometra

Using the gynecological disease screening data, univariate and multivariate analyses were performed to assess risk factors for hydrometra. As shown in **Table 1**, vaginitis was found to be highly associated with hydrometra (OR 3.81; *p* <0.001). Moreover, stratified analysis showed that trichomonad vaginitis was highly associated with hydrometra (OR 1.55), while there was no significant correlation between colpomycosis and hydrometra. Moreover, it was found that, somewhat counterintuitively, abnormal endometrial thickening and cervical polyps were protective factors for hydrometra (**Table 1**). In addition, multivariate analysis showed that advanced age, vaginitis, and ovarian cyst are factors associated with high risk for hydrometra (**Table 2**). In contrast, abnormal endometrial thickening and cervical polyp are factors that protect against hydrometra (**Table 2**). These results imply that advanced age and vaginitis are positively associated with hydrometra, and that timely treatment of vaginitis may be beneficial in reducing the prevalence of hydrometra to a certain extent.

**Table 1:** Univariate analysis of risk factors for hydrometra.

	Hydrometra (*n* = 424)	NC (*n* = 3,479)	χ^2^	*p*	*R* ^2^	95% CI
**Uterine fibroids**			3.29	0.07	0.36	0.11–1.14
Yes	3	68				
No	421	3,411				
**Prolapse of uterus**			0	0.97	0.98	0.42–2.31
Yes	6	50				
No	418	3,429				
**Adenomyosis**			0.24	0.63	0.43	0.06–3.22
Yes	1	19				
No	423	3,460				
**Cervical polyp**			25.58	<0.001	0.19	0.10–0.39
Yes	8	315				
No	416	3,164				
**Abnormal endometrium**			3.48	<0.001	0.11	0.03–0.46
Yes	2	139				
No	422	3340				
**Ovarian cyst**			1.65	0.2	1.26	0.89–1.79
Yes	39	259				
No	385	3,220				
**IUD**			0.51	0.48	0.75	0.35–1.64
Yes	7	76				
No	417	3,403				
**Trichomonad vaginitis**			6.42	0.01	1.55	1.1–2.18
Yes	43	236				
No	381	3,243				
**Colpomycosis**			0	0.99	0.63	0.08–4.83
Yes	1	13				
No	423	3,466				
**Vaginitis**			128.81	<0.001	3.81	2.99–4.87
Yes	114	306				
No	310	3,173				
**Endometrial polyp**			0.38	0.54	1.9	0.54–6.69
Yes	3	13				
No	421	3,466				

NC, women without hydrometra; R^2^, relative risk degree; IUD, intrauterine device.

**Table 2:** Multivariate analysis of risk factors for hydrometra (sample size: 3903).

	B	S.E.	Wald	*p*	Exp(B)	95% Exp(B)
Age (years)	0.11	0.01	115.25	<0.001	1.11	1.09-1.13
Uterine fibroids	-1.02	0.61	2.87	0.09	0.36	0.11-1.18
Prolapse of uterus	-0.16	0.45	0.12	0.73	0.85	0.35-2.07
Adenomyosis	-0.04	1.04	0	0.97	0.96	0.12-7.39
Cervical polyp	-1.88	0.38	24.65	<0.001	0.15	0.07-0.32
Abnormal endometrium	-2.08	0.72	8.36	<0.01	0.13	0.03-0.51
Ovarian cyst	0.44	0.19	5.3	0.02	1.56	1.07-2.27
IUD	0.25	0.43	0.32	0.57	1.28	0.55-2.98
Trichomonad vaginitis	0.61	0.2	8.91	<0.01	1.84	1.23-2.75
Colpomycosis	-1.29	1.07	1.44	0.23	0.28	0.03-2.26
Endometrial polyp	0.44	0.67	0.42	0.52	1.55	0.41-5.79
Vaginitis	1.16	0.13	74.37	<0.001	3.18	2.44-4.13
Constant	-8.67	0.62	197.28	<0.001	0	

IUD, intrauterine device

### Pathological distribution analysis of hydrometra

**Figure 2:**
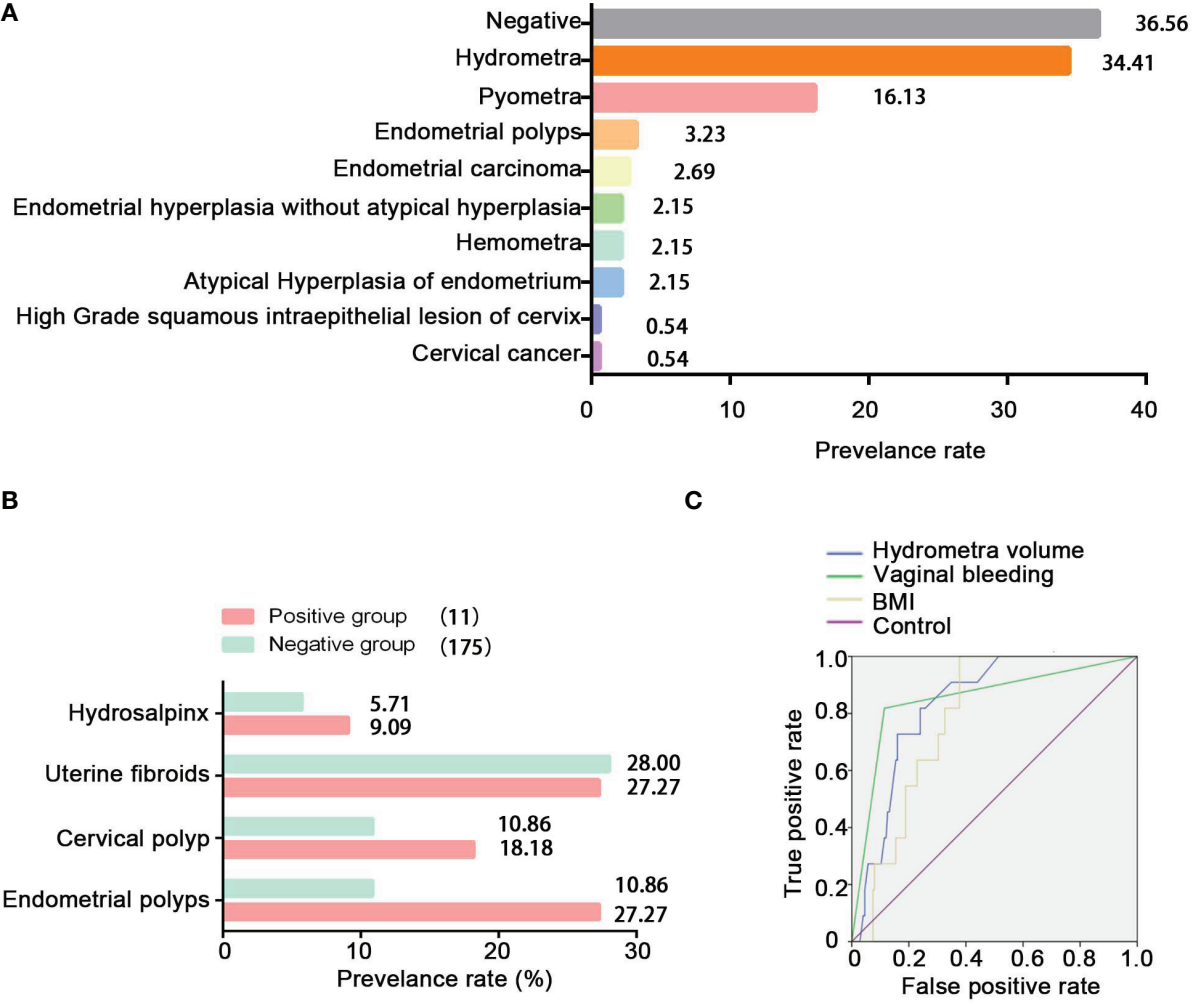
Pathological distribution of hydrometra and its predictive value in gynecological tumors. **(A)** Pathological distribution of sonographic diagnosis of hydrometra. The *y*-axis represents different pathological diagnoses, and the *x* -axis represents prevalence rate. **(B)** Comparison of hydrosalpinx, uterine fibroids, cervical polyp, and endometrial polyps between the positive group and the NC group. **(C)** ROC analysis to assess the predictive value of hydrometra volume, hydrometra complicated with BMI, or hydrometra complicated with abnormal vaginal bleeding in gynecological tumors.

To study the pathological distribution analysis of hydrometra, we studied a further 186 patients in whom hydrometra was diagnosed by sonography at Zhoupu Hospital from 1 January 2020 to 31 December 2021, and who underwent hysteroscopic operation and postoperative pathological examination. The clinical and demographic characteristics of these patients are shown in **Table S2**. The results indicate that the prevalence rate of gynecological tumors among women with hydrometra was 5.91%. Among the 186 patients, there were five cases of endometrial cancer, four cases of atypical hyperplasia of endometrium, one case of cervical cancer, and one case of high-grade squamous intraepithelial lesion of the cervix (**Figure 2A**). Moreover, the prevalence rate of inflammation was 16.23%. However, the prevalence rate of hydrometra was only 34.41%, while the negative rate was 36.56% (**Figure 2A**). These data imply that hydrometra is a high-risk factor for gynecological tumor.

### Analysis of high-risk factors of hydrometra complicated with gynecological tumors

In this study, the research data showed that the prevalence rate of hydrosalpinx, cervical polyp, or endometrial polyp was higher in the positive group than in the negative group (**Figure 2B**). Furthermore, it can be seen that BMI and hydrometra volume were higher in patients in the positive group than in those in the NC group, which implies that high BMI and hydrometra are risk factors for hydrometra complicated by gynecological tumors (**Table 3**). Further univariate analysis showed that abnormal vaginal bleeding was a high-risk factor for hydrometra to predict gynecological tumor (OR 34.88; **Table 4**). What is more, two-factor logistic regression analysis also indicated that BMI, hydrometra volume, and abnormal vaginal bleeding were high-risk factors for hydrometra to predict gynecological tumor (**Table 5**). However, diabetes mellitus, menopausal age, vaginal discharge, and menopausal years were not predictive of gynecological tumors in hydrometra patients (**Table 5**). These data indicate that hydrometra complicated by obesity, higher hydrometra volume, or abnormal vaginal bleeding is positively associated with gynecological tumors.

**Table 3:** Characteristics of patients with hydrometra.

Characteristic	Positive group (11)	NC group (175)	*t*-value	*p*
Age (years)	58.00±4.02	58.16±0.68	0.06	0.96
Number of pregnancies	2.18±0.40	2.49±0.09	0.8	0.43
Number of deliveries	1.55±0.25	1.39±0.05	0.7	0.49
Menopausal years	9.60±3.87	9.35±0.58	0.1	0.92
Menopausal age (years)	50.10±1.34	50.84±0.29	0.63	0.53
BMI (kg/m^2^)	26.23±0.57	23.76±0.21	2.96	0.003
Hydrometra volume (mm)	8.37±1.57	4.38±0.36	2.7	0.008

NC, patients without cervical cancer, high-grade squamous intraepithelial lesion of cervix, atypical hyperplasia of endometrium, or endometrial cancer; BMI, body mass index

**Table 4:** Univariate analysis of risk factors for hydrometra complicated with gynecological cancer.

	Positive group (n = 11)	NC group (*n* = 175)	χ^2^	*p*	*R* ^2^	95% CI
**Abdominal pain**			0.06	0.81	0.62	0.13–2.99
Yes	2	46				
No	9	129				
**Vaginal discharge**			0.02	0.89	2.82	0.31–25.70
Yes	1	6				
No	10	169				
**Vaginal bleeding**			33.8	<0.001	34.88	7.03–172.98
Yes	9	20				
No	2	155				
**Menopause time**			0	1	1.59	0.2–12.98
Yes	10	151				
No	1	24				
**Diabetes mellitus**			0	1	1.65	0.19–14.2
Yes	1	10				
No	10	165				
**Hypertension**			0.54	0.46	2.25	0.56–9.06
Yes	3	25				
No	8	150				
**Intrauterine occupation**			1.33	0.25	3.08	0.75–12.61
Yes	3	19				
No	8	156				
**Uterine fibroids**			0	1	0.96	0.25–3.79
Yes	3	49				
No	8	126				
**IUD**			1.01	0.32	1.19	1.12–1.27
Yes	0	28				
No	11	147				

NC, patients without cervical cancer, high-grade squamous intraepithelial lesion of cervix, atypical hyperplasia of endometrium, or endometrial cancer; IU, intrauterine device; R^2^, relative risk degree.

**Table 5:** Multivariate analysis for risk factors of hydrometra complicated with gynecological cancer (sample size: 186).

	B	SE	Wald	P	Exp(B)	95% Exp(B)
Age (years)	–0.38	0.27	1.96	0.16	0.68	0.40–1.17
Diabetes mellitus	0.53	4.26	0.02	0.9	1.71	0.00–7,171.71
Hypertension	2	1.6	1.56	0.21	7.38	0.32–169.98
Menopausal age	0.16	0.1	2.74	0.1	1.18	0.97–1.42
Abdominal pain	–0.61	1.33	0.21	0.65	0.55	0.04–7.34
Vaginal discharge	2.37	2.43	0.96	0.33	10.71	0.09–1,240.55
Hydrometra volume (mm)	0.29	0.12	6.09	0.01	1.33	1.06–1.68
Menopausal years	0.4	0.27	2.21	0.14	1.49	0.88–2.52
Vaginal bleeding	6.12	1.93	10.09	0.001	452.47	10.40–1,9682.23
IUD	–21.89	5330	0	1	0	0
Uterine fibroids	–0.25	1.14	0.05	0.83	0.78	0.08–7.35
BMI (kg/m^2^)	0.71	0.34	4.45	0.04	2.04	1.05–3.97
Constant	–14.42	13.32	1.17	0.28	1	

IUD, intrauterine device.

### Analysis of the predictive value of hydrometra in gynecological tumors

To study the predictive value of abnormal vaginal bleeding, hydrometra volume, and BMI in gynecological tumors, receiver operating characteristic (ROC) curve analysis was performed. The area under the curve (AUC) was used to assess the sensitivity of different risk factors. The results showed that the AUC of hydrometra complicated by abnormal vaginal bleeding (0.852) was higher than the AUC of hydrometra volume (0.834), while the AUC of hydrometra complicated by obesity (0.784) was the lowest of the three factors (**Figure 2C**). We also considered the cut-off value of hydrometra volume and BMI in gynecological tumor prediction. The results showed that, at a cut-off value for hydrometra volume > 4.75 mm, the diagnostic sensitivity was 81.8% and the false-positive rate was 24%, with the largest difference value between the sensitivity rate and false-positive rate. We also found that, when a BMI > 23.92 kg/m^2^ was taken as the cut-off value, the diagnostic sensitivity was 100% and the false-positive rate was 37.7%, with the largest difference value between the sensitivity rate and false-positive rate. Furthermore, when abnormal vaginal bleeding was considered as a gynecological tumor predictor factor, the diagnostic sensitivity was 81.8% and the false-positive rate was 11.4%. These data imply that a hydrometra volume >4.75 mm, hydrometra associated with a BMI > 23.92 kg/m^2^ , and hydrometra associated with abnormal vaginal bleeding are all of predictive value for gynecological tumors.

## Discussion

Hydrometra is a common gynecological disease. For a long time, it was regarded as only a benign disease, without recognizing its harmfulness and value in the tumor prediction. In this study, we observed that the prevalence rate of hydrometra was 10.86%, similar to the rate reported by Bar-Hava etal. (1). Moreover, the prevalence rate of cancer and precancerous lesions among hydrometra patients was 5.91%. These data suggest that hydrometra is not only a benign disease, but also an important manifestation of gynecological tumors. Therefore, hydrometra cannot be ignored, and should receive more attention.

Many studies have investigated the cause of hydrometra. Antonson etal. (23) reported that increased serum estrogen levels resulted in hydrometra in mice. Moreover, increased levels of lactoferrin, complement C3, and chitinase 3-like 1 (CHI3L1) have been found to be associated with hydrometra (24). López Rivero etal. (25) reported that an isthmocele due to a cesarean section was also associated with persistent hydrometra. In addition, abnormal development of the urogenital tract has been reported to be a cause of hydrometra (26). In addition, McQueen etal. (27) reported that increased triggering receptor expressed on myeloid cells 1/3 (TREM-1/3) infected mice through promoting transepithelial neutrophil migration in the uterus and uterine glands. However, most current studies have focused on animals, with limited data from humans. Therefore, high-risk factors for hydrometra development in humans are still unknown. In this study, advanced age, vaginitis, and ovarian cyst were found to be high-risk factors for hydrometra. It is speculated that elderly women with uterine atrophy and degeneration are more likely to experience tract obstruction, which contributes to hydrometra, and that vaginitis, which is prone to upward spread, can also induce hydrometra. Similarly, ovarian cysts may affect fallopian tube peristalsis and reduce patency, thus inducing hydrometra. However, it is difficult to believe that abnormal endometrial thickening and cervical polyps, which are thought to reduce the patency of the cervix and uterine cavity, could protect against hydrometra, as found in this study. This requires further investigation.

Inflammation is another important cause of hydrometra. Yeung etal. (28) reported that pyometra accounted for 47.8% of cases of intrauterine fluid in 228 patients, with hydrometra accounting for 43.0% and hematometra for the remaining 9.2%. However, these findings differ from our data. In our study, we found that pyometra accounted for only 16.13% of cases of intrauterine fluid in 186 patients, whereas hydrometra accounted for 34.41% and hematometra for 2.15%. These results might be ascribed to differences between regions and different populations. These data suggest that inflammation is not the most important cause of hydrometra, which would explain the fact that many patients with hydrometra do not respond to anti-inflammatory therapy in clinical practice. Moreover, Sik Wing Yeung etal. (28) found that advanced age (>75 years) was an independent risk factor for pyometra, whereas we found no independent risk factor for pyometra.

Other studies have reported that further factors associated with endometrial cancer, notably an endometrial stripe ≥ 2 cm, higher body mass index and waist-to-hip ratio, excessive unopposed exposure of the endometrium to estrogen, increasing age, obesity, hypertension, diabetes mellitus, and abnormal gene expression (**Table S3**) (29–36). In addition, potential risk factors for cervical cancer include human papillomavirus infection, a family history of cancer, vaginal bleeding, hypertension, multiple sexual partners, initiation of sex at a young age, smoking, and hormonal contraceptive use (**Table S4**) (37–43). However, the role of hydrometra in endometrial cancer and cervical cancer is still unknown. In this study, it was found that hydrometra was a risk factor for endometrial cancer and cervical cancer. We believe that this is a new discovery. More importantly, it was found that higher hydrometra volume, higher BMI, and abnormal vaginal bleeding, when complicating hydrometra, are potential predictive factors for endometrial cancer and cervical cancer. This finding is of clinical value and warrants further study.

However, our study had some limitations. For example, owing to the relatively small sample size, multivariate analysis result in high odds ratios and wide 95% CIs. Therefore, our results need to be validated in a large sample. In addition, the study was retrospective and was carried out in a single center. The results should be confirmed in multicenter prospective studies.

## Conclusions

In conclusion, hydrometra is a common symptom in women, especially in advanced age women. Advanced age, vaginitis and ovarian cyst have found to be responsible for hydrometra, while anti-inflammation might be beneficial to prevent the occurrence of hydrometra. Moreover, hydrometra is a risk factor for endometrial cancer and cervical cancer, especially with higher hydrometra volume, higher BMI, and abnormal vaginal bleeding. Therefore, timely hysteroscopy and histopathological examination for hydrometra patients will be beneficial for early detection of endometrial and cervical tumors, with important clinical value.

## Data availability statement

The original contributions presented in the study are included in the article/**Supplementary Material**. Further inquiries can be directed to the corresponding authors.

## Ethics statement

The studies involving human participants were reviewed and approved by Zhoupu Hospital Ethics Committee. The patients/participants provided their written informed consent to participate in this study.

## Author contributions

Study Design: JFW and SHW; Data Interpretation: JFW, SQW,and ZL; Manuscript Preparation: JFW and SHW; Literature Search: LZ and SHW; Funds collections: JFW. All authors contributed to the article and approved the submitted version.
