# Supplementary material for: Epidemiological analysis of hydrometra and its predictive value in gynecological tumors
Source: Front Oncol. 2023 Jan 5;12:1028886. doi: 10.3389/fonc.2022.1028886 (PMC9851649; doi:10.3389/fonc.2022.1028886)
Supplement: Supplementary file 1 [file DataSheet_1.zip › Supplementary Table (1).DOCX]

#### **Table S1**. Clinical and demographic characteristics for 3903 gynecological screening population

|  | Hydrometra  (424) | NC  (3479) |
| --- | --- | --- |
| **Age**  ≤50  51~60  61~70  ≥71  **Uterine fibroids**  **[Prolapse](javascript:;) [of](javascript:;) [uterus](javascript:;)**  **[Adenomyosis](javascript:;)**  **[Cervical](javascript:;) [polyp](javascript:;)**  **Abnormal endometrium**  **[Ovarian](javascript:;) [cyst](javascript:;)**  **IUD**  **[Trichomonad](javascript:;) vaginitis**  **Colpomycosis**  **Vaginitis**  **[Endometrial](javascript:;) [polyp](javascript:;)** | 12  142  270  0  3  6  1  8  2  39  7  43  1  114  3 | 376  1793  1304  6  68  50  19  315  139  259  76  236  13  306  13 |

NC: Women without hydrometra；IUD: Intrauterine device
