# Supplementary material for: Epidemiological analysis of hydrometra and its predictive value in gynecological tumors
Source: Front Oncol. 2023 Jan 5;12:1028886. doi: 10.3389/fonc.2022.1028886 (PMC9851649; doi:10.3389/fonc.2022.1028886)
Supplement: Supplementary file 1 [file DataSheet_1.zip › Supplementary Table (2).DOCX]

#### **Table S2**. Clinical and demographic characteristics for 186 hydrometra

|  | Positive group  (11) | NC group  (175) |
| --- | --- | --- |
| **Age**  **Number of pregnancy**  **Number of delivery**  **Menopausal years**  **Menopausal age**  **BMI index**  **Hydrometra volume**  **Caesarean section**  **Number of menopause patients**  **Tubal sterilization**  **Vaginal discharge**  **Vaginal bleeding**  **Diabetes mellitus**  **Hypertension**  **Intrauterine occupation**  **Uterine fibroids**  **IUD**  **Hydrosalpinx**  **Cervical polyp**  **TCT(≥ASCUS)**  **HPV infection**  **Pathologic distribution**  Normal  EHWAH  Pyometra  Hydrometra  Hemometra  Endometrial polyp  Endometrial cancer  AHE  Cervical cancer  HSIL | 58.00±4.02  2.18±0.40  1.55±0.25  9.60±3.87  50.10±1.34  26.23±0.57  8.37±1.57  0  10  1  1  9  1  3  3  3  0  1  2  1  0  0  0  0  0  0  0  5  4  1  1 | 58.16±0.68  2.49±0.09  1.39±0.05  9.35±0.58  50.84±0.29  23.76±0.21  4.38±0.36  23  151  13  6  20  10  25  19  49  28  10  19  4  15  67  4  30  64  4  6  0  0  0  0 |

NC: Patients without cervical cancer, high grade squamous intraepithelial lesion of cervix, atypical hyperplasia of endometrium and endometrial cancer; IUD: Intrauterine device; BMI: Body mass index; EHWAH: Endometrial hyperplasia without atypical hyperplasia; AHE: Atypical hyperplasia of endometrium; HSIL: High grade squamous intraepithelial lesion of cervix
