# Supplementary material for: Epidemiological analysis of hydrometra and its predictive value in gynecological tumors
Source: Front Oncol. 2023 Jan 5;12:1028886. doi: 10.3389/fonc.2022.1028886 (PMC9851649; doi:10.3389/fonc.2022.1028886)
Supplement: Supplementary file 1 [file DataSheet_1.zip › Supplementary Table (3).DOCX]

#### **Table S3**. Risk factors for endometrial cancer

| Author | Risk factors | Published year |
| --- | --- | --- |
| Cavaliere AF et al.  Michael M Braun et al.  Piergentili R et al.  Monica Hagan Vetter et al.  Yu Gao et al.  Michelle T Doherty et al.  Olivia Raglan et al.  Ellen Stelloo et al. | Genetic predisposition(Abnormal expression of LncRNA, such as AL161431.1, C2orf48, etc. )  Unopposed estrogen therapy, early menarche, late menopause, tamoxifen therapy, nulliparity, infertility or failure to ovulate, PCOS, age older than 50 years, hypertension, DM, obesity, thyroid disease, and Lynch syndrome  Genetic predisposition(Abnormal expression of LncRNA, such as ABHD11-AS1, AL161431.1, etc. )  Endometrial stripe of ≥2 cm  Smoking and HRT are risk factors, while coffee intake and high BMI are protective factors  Atypical and non-atypical endometrium hyperplasia  Higher Body mass index and waist-to-hip ratio  p53-mutant and MSI | 2021  2016  2021  2020  2022  2020  2019  2016 |

PCOS: Polycystic ovary syndrome; DM: Diabetes mellitus; HRT: hormone replacement therapy; MSI: Microsatellite instability; ABHD11-AS1: ABHD11 antisense RNA 1; C2orf48: Ribonucleotide reductase regulatory subunit M2
