# Supplementary material for: Epidemiological analysis of hydrometra and its predictive value in gynecological tumors
Source: Front Oncol. 2023 Jan 5;12:1028886. doi: 10.3389/fonc.2022.1028886 (PMC9851649; doi:10.3389/fonc.2022.1028886)
Supplement: Supplementary file 1 [file DataSheet_1.zip › Supplementary Table (4).DOCX]

#### **Table S4**. Risk factors for cervical cancer

| Author | Risk factors | Published year |
| --- | --- | --- |
| Pimple S et al.  [Steven B Holloway](https://pubmed.ncbi.nlm.nih.gov/?term=Holloway+SB&cauthor_id=33606367) et al.  Lise M A De Strooper et al.  [Bo T Hansen](https://pubmed.ncbi.nlm.nih.gov/?term=Hansen+BT&cauthor_id=34412617) et al.  Becker TM et al.  Castle PE et al.  McIntyre-Seltman K et al. | Persistent infection with high-risk HPV, high parity, long-term use of oral contraceptive pills, tobacco consumption, co-infection with other sexually transmitted agents, lifestyle factors such as multiple sexual partners, younger age at first sexual intercourse, immunosuppression  Abnormal gene expression(NTD-N3)  Abnormal gene expression(FAM19A4/ mir124-2 methylation)  Regional differences，earlier sexual debut, more partners and higher prevalence of ever having had a STI  Smoking  Injectable hormonal contraceptive use  Smoking | 2022  2021  2018  2021  1994  2005  2005 |

HPV: Human papillomavirus; NTD-N3: Necrotic tumor debris N3; STI: sexually transmitted infection; FAM19A4: Family with sequence similarity 19 member A4, C-C motif chemokine like
